# Poverty and mental health: the work of the female sanitary inspectors in Bradford (c. 1901–1912)

**DOI:** 10.1057/s41599-018-0128-2

**Published:** 2018-06-19

**Authors:** Pamela Dale

**Affiliations:** 1 University of Exeter, Exeter, UK

## Abstract

Although there are many excellent studies of the work of pioneer women public health officers, few accounts dwell on mental health issues or discuss any relationship that such staff might have understood to exist between poverty and mental health in the early twentieth century. This is a remarkable omission considering that social and feminist historians have highlighted the problems created by the way early practitioners sought to manage poverty and arguably the poor. Drawing on records created by Female Sanitary Inspectors (FSIs) in Bradford, this study chronicles distressing economic and social conditions but also reveals encounters between the staff and people experiencing mental health problems and mental health crises. The ways in which the FSIs chose to both make and deny links between the abject poverty witnessed in the slum districts and cases of mental disorder forms an important strand to the analysis that follows. Interestingly, it is the well-being of the staff that emerges as a persistent and even over-riding concern.

## Introduction

The history of public health in the UK tends to be divided into two distinct phases, with John Welshman (2000, pp. 26–27), amongst others, highlighting the contrast between nineteenth-century sanitary reform and the development of personal and preventative health services in the twentieth century. He emphasises the way Victorian sanitary reform continues to be presented as a ‘heroic struggle’, whilst accounts of later public health schemes tend to pessimistically focus on the decline of public health as a ‘medical specialism’ and ‘local government responsibility’. Over the last two decades a revival of interest in municipal medical projects (Welshman, 2000; Harris, 2004, pp. 219–242; Levene and colleagues 2011) has started to correct this picture, at least for the interwar period. Yet, the idea that nothing very interesting was happening in the public health sphere has been continuously challenged by studies concerned with the early-twentieth-century proliferation and evolution of services ‘for mothers and infants, provision for schoolchildren, and individual diseases such as tuberculosis’ (Welshman, 2000, pp. 26–27).

The distinctive contribution of women officers to these services has been widely recognised (Dingwall et al., 1988, p. 187; Davies, 1988). From the 1890s, there was gradual but significant expansion in the number of women employed in public health work across the UK, although there were different routes into the sector and job titles and duties showed considerable variation. According to Jennifer Haynes, ‘the overlapping roles of woman sanitary inspector, health visitor and tuberculosis visitor were performed alongside each other in local public health departments, often by the same women’ (Haynes, 2006, p. 15). The historiography has also been particularly concerned with the emergence of the woman sanitary inspector (Brimblecombe, 2000) and her reinvention as a health visitor (Dingwall et al., 1988, pp. 173–203) whose identity became increasingly bound up with that of the nurse. Anne Kelly and Anthea Symonds (2003) certainly develop important insights from considering the long-term evolution of health visiting as a branch of community nursing. However, most controversy and debate still centres on the origins of the profession and the ‘process by which health visiting was captured as a branch of nursing’ (Dingwall et al., 1988, p. 188).

Many excellent studies have showcased the work of pioneer women sanitary inspectors (Brimblecombe, 2000; Haynes, 2006), and the later profession of health visiting (Clark, 1973; Owen, 1977; Davies, 1988; Welshman, 1997; Malone, 2000). Unfortunately, few accounts dwell on mental health issues or discuss any relationship that staff might have understood to exist between poverty and mental health in the early twentieth century. This is an important omission since poverty has been a major theme in the historiography. Social and feminist historians have highlighted the problematic relationship early practitioners had to the management of poverty (Davin, 1978; Dyhouse, 1979; Ross, 1993, pp. 204–209; Kelly and Symonds, 2003, pp. 16–20). They suggest that this exacerbated tensions between practitioners’ care and control functions. These served to restrict both professional development and the effectiveness of services offered (Lewis, 1986, pp. 109–115).

Using records created by, and for, the Female Sanitary Inspectors (FSIs) in Bradford c. 1900–1912, this case study finds no shortage of reports describing distressing economic and social conditions. It also uncovers some personal notes about unplanned encounters between the professional women and people experiencing mental health problems and mental health crises. The study explores how the FSIs chose to both make and deny links between the abject poverty witnessed in the slum districts and cases of mental illness. Interestingly, the well-being of the staff emerges as a persistent and even over-riding concern as sanitary inspection was combined with, and then increasingly displaced by, health visiting duties.

## Background

Health visitors today routinely seek to promote mental health, as well as support those with mental health problems. Such is the range of current activities that some leading practitioners are explicitly reminding staff, and employers, that their primary focus should remain on supporting young families (Adams, 2012). The specific health visitor concern with new babies, small children, and their main carers harkens back to the earliest days of the infant welfare movement and sits comfortably within both critical and celebratory histories of the profession (Dwork, 1987, pp. 226–230). The evolution of mental health work is arguably less well understood. Modern practitioners tend to date the major expansion, if not origins, of these duties to the late 1960s and early 1970s. Textbooks from this era include introductions to ‘psychiatric aspects of the family’, as well as ‘special aspects of psychiatry’ (Howells, 1967). This timeline draws attention to the impact of the Jameson Report (1956); the gradual switch to GP attachment; the reorganisation of local authority services and the NHS (1972–1974); the creation of the Council for the Training of Health Visitors (1962); and the development of new principles of health visiting by the renamed Council for the Education and Training of Health Visitors, 1977–1982 (CETHV, 1982) that underpinned revised training schemes (Dingwall, 1977, pp. 187–188).

Yet, health visitor engagement with mental health and mental illness had much longer antecedents that merit exploration. Until 1974, women public health officers (including health visitors and women employed as sanitary inspectors but performing health visiting duties) served under the direction of individual local Medical Officers of Health (MOsH) and their work was strongly influenced by his changing priorities over many decades. MOsH tended to take a holistic view of health and routinely discussed the causes and consequences of ill-health as part of their statutory public health duties and responsibilities for overseeing municipal health services (Newsholme, 1936). The creation of the National Health Service (NHS) in 1948 and the loss of municipal general hospitals encouraged MOsH to develop a new focus on promoting mental health; preventing mental illnesses; and offering diagnostic, treatment, and support services for patients and their families. John Welshman describes some innovative local schemes but notes that their potential was restricted by a lack of support from central government (Welshman, 2000, pp. 222–230). Nevertheless, health visitors performed a variety of roles within such programmes. These were particularly strong in parts of Scotland where, for example, multi-disciplinary psychogeriatric services were developed in the 1950s and 1960s (Nottingham and Dougall, 2007). Some former MOsH regarded the period 1948–1974 as the true ‘golden age’ of public health services (Francis, 1987, p. 141), but older colleagues reminded them that they, and their staff, enjoyed even greater powers and responsibilities in the years 1929–48 when the relationship between poverty and health came under ever greater scrutiny (Kelly and Symonds, 2003, pp. 34–35).

The 1929 Local Government Act gave local authorities new responsibilities for mental health services. The Ministry of Health encouraged detailed planning and supported ambitious MOsH who wanted to develop innovative schemes in response to the 1930 Mental Treatment Act (MTA). There are some examples of health visitors supporting these activities by liaising with and between different clinics; providing background details about patients and families; visiting discharged patients; and working with families receiving ongoing support because of long-term sickness or disability. While health visitor involvement with individual patients experiencing acute mental illness was extremely limited in the first half of the twentieth century, the interwar period saw the profession accepting increasing responsibilities for the long-term support of mentally frail older people and certain other patient groups. More important, however, was the health propaganda work health visitors were tasked with. Health visitors offered individual and group health education, which extended to the promotion of mental well-being, and also sign-posted people towards services designed to offer the benefits of early treatment following the 1930 MTA. Interwar health visitors were often well-versed in the principles of the mental hygiene movement (Thomson, 2006, pp. 48–49, p. 170, pp. 175–176, pp. 191–194, p. 239), and in some areas staffed key parts of the services established to implement the 1913 Mental Deficiency Act (Thomson, 1998).

MOsH had always encouraged their staff to take a holistic view of health and this was supported by the development of ever more comprehensive and inclusive health services at a local and national level (Levene and colleagues, 2011), as well as the academic study of social medicine (Mckeown and Lowe, 1974). Yet, even before 1929, health visitors had planned and unplanned involvement with mental health issues as an inevitable consequence of their work supporting infant welfare programmes, school medical inspections and clinics, tuberculosis schemes, and venereal disease services. The routine nature of these activities means that they typically received little attention in contemporary reports of interwar health visiting written by MOsH beyond praise for the tact trained health visitors could be relied upon to use to diffuse difficult situations or cope with distressing cases. Such records provided no space for health visitors to recount their personal experiences, although MOsH sometimes explained high levels of staff sickness with reference to the pressure a particular team had been under. This silence can usefully be contrasted with the way an earlier generation of female officers used their independently authored reports to describe their experiences of working with a wide range of client groups and explicitly theorise about the causes and consequences of mental illness. Poverty is a consistent theme, and major concern, running through the Edwardian Bradford FSI reports.

## The Bradford FSI reports

The *Report of the Work of Female Sanitary Inspectors 1902*–*1911* provides a useful guide to the activities and opinions of pioneer women public health officers in Bradford. The FSIs worked out of the public health department which was led by the Bradford MOH. However, the FSIs carefully guarded their independence and made a point of making personal reports to the health committee. Their quarterly reports were addressed to ‘Mr Chairman and Gentlemen’. The first FSI signed her own reports, and once a team of staff were assembled, the senior FSI reported on their behalf. FSI reports tended to increase in length and complexity, except on occasions when pressure of work was used as an explanation for a delayed or abbreviated submission. The content of, and audience for, the reports also underwent subtle changes. The MOH became a more visible actor as the FSIs undertook special projects on his behalf. Reports also highlighted instances where the MOH had explicitly responded to discoveries, and requests, originally made by the FSIs. At other times the FSI reports shifted from simply detailing key findings to exhorting the health committee (and other actors and agencies) to do more to resolve long-standing problems within the city. These appeals were also addressed to a wider public, as the reports were printed for distribution and received press coverage.

The FSI reports offered an opportunity to showcase the work of women public health officers. There was a self-consciousness about the way the Bradford FSIs presented themselves. They emphasised that they were cognisant with the latest scientific and medical thinking.^1^ Yet, they also claimed that their experiences allowed them to offer unique insights into a variety of social problems. Jennifer Haynes (2006), using examples mostly drawn from the London area, shows how other women engaged in Edwardian public health work used similar strategies to publicise themselves and their work in an effort to advance the causes of both. There was a determined effort to open up more career opportunities for women and achieve/retain equality with male officers. The staff Haynes describes performed duties similar to those undertaken by the FSIs in Bradford, and with growing responsibilities came the risk of excessive workloads and the misdirection of available labour. Indeed, so great were the range of actual and potential duties that resources and expertise were necessarily stretched (Dale and Mills, 2007, p. 120). This raised questions about the appropriate training, recruitment, and deployment of staff.

The main duty of the Bradford FSIs, at least initially, was house-to-house visiting in the worst slum districts with the aim of suppressing nuisances and reducing overcrowding. Their reports include long lists of environmental hazards and property defects, and descriptions of the worst cases of overcrowding (with the FSIs personally measuring the size of available accommodation and dividing this measure of space between the number of usual occupants).

The measurement of overcrowded houses is at times accomplished under considerable difficulty. The height of the lower portions of several garrets has been under four feet, and at this time of year the walls are alive with bugs, which crawl about even in daylight…one is either unpleasantly near the walls or the beds. When to this is added a heavy, sickly atmosphere…. (FSIR 30 June 1906, pp. 8–9).

The early Bradford FSIs were, like their male colleagues, qualified sanitary inspectors and this training shaped their evolving duties. Dingwall et al. (1988, p. 186) note that they were ‘competent to inspect cattle markets, slaughterhouses, sewage disposal and similar offensive environments’ but suggest there was always pressure to use the FSIs differently, to address more ‘feminine matters’ such as homes, infants, and midwives. Such a narrowing of concerns threatened the ambitions of women public health officers. The defensive action fought by the Women Sanitary Inspectors’ Association is described by Dingwall et al. (1988, p. 187). It is hard to escape their conclusion that the employment of women as health visitors, roles requiring less training and not offered on terms of equality with male officers, marked a significant setback. Jennifer Haynes (2006) offers a slightly more nuanced account that tends to downplay cost considerations to concentrate on changing employer requirements. She usefully highlights a period of coexistence, when posts were available for women as sanitary inspectors and health visitors. Nevertheless, she readily accepts the main conclusions outlined above about the dilution of skills and the consequent downgrading of the profession. The evolving duties of the Bradford FSIs reflect these national debates but at this moment of professional transition there was still scope for the staff to make independent statements about various aspects of their work.

It was the requirement to gain access to each property (and each separate household within it) and interview the residents that led to the planned and unplanned encounters between the staff and individuals living with physical and mental illnesses and/or exhibiting various kinds of challenging behaviours. At first vignettes of typical and a-typical cases were included in the reports to familiarise the health committee with the work of the FSIs and support calls for more staff and better working conditions. Over time, however, the presentation of cases (some followed up over several months or even longer) became more systematic and the FSIs started to theorise about the social and economic problems experienced by the slum dwellers with an emphasis on the relationship between poverty and ill-health (Mills and Dale, 2007). Concerns about physical health, and differential mortality and morbidity rates, are easily distilled from FSI reports discussing tuberculosis cases and investigations into infant deaths. Anxieties about mental health are both ever present and more elusive.

Haynes, who has written about various groups of pioneer women public health officers, notes that it is very difficult to develop full biographies of the staff.^2^ But the Bradford FSI reports, considered here, made much of the fact that all the women appointed were qualified sanitary inspectors, and in some cases mentioned additional training or hospital experiences that added value to their contribution. Miss E H Jones, first appointed in 1904 and promoted to Chief Woman Inspector later that year, described herself as an Associate of the Royal Sanitary Institute and Diplomée of the National Health Society. Yet, in common with the women sanitary inspectors in London (Haynes, 2006), the Bradford FSI reports dwelt on the personal qualities of the staff. In an appeal for more, and better paid staff, Miss Jones recorded:

Since my last report… Miss Dawson has been appointed to Lambeth. Her departure was an irreparable loss to this department. Her four years’ knowledge of the inhabitants of certain districts was invaluable, and her devotion to the work, and conscientiousness in carrying out its duties, cannot be too highly praised. The work is in itself of a particularly difficult nature, involving as it does, so much teaching under most adverse surroundings. It requires a good deal of knowledge and skill, allied to inimitable tact… (FSIR 30 September 1908, p. 14).

For Haynes, an emphasis on behaviour and character was a defensive strategy designed to conceal the deficiencies of the ‘education’ received by some officers prior to training, and the limitations of available training courses (Haynes, 2006, pp. 93–115). In Bradford, these issues were complicated by salary differentials that encouraged the most ambitious staff to move to London to develop their careers. For instance, it is probable that Miss Carey (who made a fleeting appearance as an assistant inspector in the Bradford records 1903–1904) went on to become the leading professional figure described by Haynes (Haynes, 2006, p. 76, p. 123, p. 140).

Bradford staff ruefully noted the discrepancy in salaries, and opportunities, available to their female colleagues in the capital (FSIR 30 September 1904, p. 8; 30 September 1908, p. 14). They also carefully followed developments in neighbouring areas, as Yorkshire was home to many pioneer schemes involving women public health officers. Several of its urban centres were early appointers of women sanitary inspectors (Haynes, 2006, p. 48). In 1907, Sheffield was the first local authority to insist that its women officers were triple qualified with certificates in nursing and midwifery, as well as from the Royal Sanitary Institute (McIntosh, 1998). However, the Bradford FSIs seemed particularly interested in developments in Leeds. They wrote approvingly of innovations (particularly in relation to staff training, numbers, and salaries) in that city (FSIR 31 March 1907, p. 6; 30 September 1907, pp. 6–7). Dr Cameron, the Leeds MOH, was a notable supporter of equality between male and female sanitary inspectors (Dingwall et al., 1988, p. 186). Interestingly, the FSI reports make no mention of other local schemes that employed women as health visitors as an alternative to having sanitary inspectors or a combined post. That said, Huddersfield’s schemes are prominent in contemporary and historical accounts of the infant welfare movement (Marland, 1993), and neighbouring Halifax endured very public wrangles over the appointment of female staff (Minutes of the Halifax Health Committee and Halifax MOH reports 1905–1909).

## Ideas about poverty

In the districts where the Bradford FSIs were deployed, poverty was endemic. According to one description:

In one overcrowded home there was no bedstead of any description, the only bedding being an old straw mattress on which slept two parents and seven children, their only covering the old clothes worn during the day (FSIR 31 March 1909, p. 15).

Yet the attention given to describing unusual homes (often identified by a street name) completely empty of food, furniture, and bedding suggests that such extreme distress was seen as a problem separate from the day-to-day struggles of the majority of slum dwellers. The FSIs were initially tasked with monitoring housing conditions (Dale and Mills, 2007) but descriptions of people’s accommodation problems made no sense without reference to a whole range of other topics. For example, a discussion about the danger of working mothers revealed that the FSIs visited 200 mothers of newborns who worked in 1908–1909; 28 of these babies died (including 15 belonging to the 70 unmarried mothers in the cohort). The FSIs described the ‘evils’ of the practice and noted that the majority of the women worked:

…out of sheer necessity, because the wages of the husband will not meet the needs of the family. During the past winter the distress would have been more acute but for the assistance rendered by the wife (FSIR 31 March 1909, p. 10).

The FSIs believed they were making vital discoveries. Yet, they were also clearly tailoring their reports to key audiences in Bradford where an alliance of radical and socialist politicians and reforming officials was beginning to transform the scope and delivery of local services (Dale and Mills, 2007, p. 114). Bradford owes much of its reputation as an innovative Edwardian local authority to initiatives aimed at improving child welfare through a number of school-based programmes (Harris, 1995, p. 21) which the FSIs had limited involvement with. There was, however, significant progress by statutory and voluntary agencies (Bradford Council of Social Service, 1923) in other areas. The FSIs were involved with organising charitable assistance for the aged poor and making visits to tuberculosis patients (to maximise their comfort, encourage home hygiene to avoid infecting family members, and disinfect properties after deaths). More significant still were schemes to not just inspect midwives but improve standards of midwifery (Dale and Fisher, 2009).

Infant and maternal welfare issues are conspicuously absent from the first FSI reports but became increasingly important over time. In Bradford the FSIs led an investigation into infant deaths and then began a wider survey of infant feeding practices. They then began a systematic programme of visits to all newborns in the poorer districts. The services that the FSIs wanted to develop to meet the obvious needs of poor women and their families were very slow to develop before 1911. Staff contrasted the willingness of the council to feed poor school-children with their reluctance to extend such schemes to younger children or pregnant and lactating women.

Few people, especially men, seem to realise that infants are alive before birth, and therefore the *expectant* mother should be the first to be considered. For the future of the race, it is high time that the community seriously considered this question, for unless better wages can be provided for the men, it is necessary to consider the desirability of feeding the poor expectant mother, and providing necessitous weakly infants with suitable nourishment (FSIR 1 October 1909–1930 September 1910, pp. 11–12).

In the absence of municipal programmes, the FSIs did much to stimulate and direct the work of the Cinderella Club,^3^ aided by the larger and more influential Guild of Help (Laybourn, 1994).

The FSIs praised and encouraged these new voluntary sector activities, as well as municipal schemes, but also pointed out their limitations and the need for yet more radical action. A critique of the local operation of the Poor Laws, with a focus on the unhelpful attitude adopted by its officials, was a developing theme in the FSI reports (Dale and Mills, 2007). Increasingly, local issues were contextualised with reference to national debates about social and economic problems. The reports also engaged with the key themes addressed by major public health conferences of the period (with papers cited and summarised in the FSI reports). The Inter-departmental on Physical Deterioration, which reported in 1904, brought together a number of long-standing, but previously disparate, concerns about the health of the nation and the problems of urban and industrial life more generally. Its call for environmental improvements and public health reforms found an obvious echo in FSI reports before and after its publication.

It is noticeable, however, that the tone of the FSI reports changed over time. In their important study of community nursing, Kelly and Symonds (2003, p. 17) hypothesise that as the state took increasing responsibility for the poor this set up a number of ‘ideological contradictions’, that people working in the sphere of managing poverty would have been acutely aware of and personally affected by. The FSIs certainly became convinced that advice-giving was only going to be effective if the statutory and/or voluntary sectors could provide the poor with at least the minimum resources necessary to implement it (FSIR 30 September 1908: 8–9; Lewis, 1986, pp. 109–115). This shift from nineteenth-century ideas about managing poverty to a distinctly twentieth-century concern with eradicating it was, however, complicated by the way discourses about both imperialism and motherhood became linked to the management of poverty (Kelly and Symonds, 2003, pp. 16–19; Davin, 1978).

All three strands shaped, and were in some ways exemplified by, the new profession of health visiting. This was a term used with increasing frequency in the FSI reports (1 October 1909 to 1930 September 1910, p. 10). It was in response to professional difficulties (both in relation to employers and clients) that the FSIs explicitly called attention to their official status and specialist scientific knowledge. This was a way of downplaying the gendered nature of their work and the tensions inherent in a system that required working-class families to accept, even welcome, intrusive middle-class lady visitors into their homes for the express purpose of making official assessments of standards of housekeeping and childcare (Davin, 1978; Dyhouse, 1979; Lewis, 1984, pp. 38–40; Ross, 1993, pp. 204–209, Vincent, 1991, pp. 34–35, Dale, 2008, p. 70). Demonstrating familiarity with key publications was an important part of this professional strategy. Women public health officers employed in Bradford were not, however, just passive consumers of material such as the findings of the 1904 Inter-departmental Committee. They intended to make a personal and professional contribution that aimed to influence local and national government policies. Staff also tried to raise public consciousness about the problems of slum life. The aim was to attract funds for specific charitable projects and, perhaps more importantly, encourage support for the wide-reaching reforms and expanded municipal schemes favoured by the FSIs.

The FSI reports were written within a distinctive public health tradition. They are both similar to, and different from, reports compiled by MOsH. There was certainly agreement on the major topics of concern. Indeed, by the end of the study period the FSIs were eager to demonstrate how they were advancing agendas and inquiries explicitly initiated by their MOH. However, FSIs in Bradford, and women sanitary inspectors elsewhere (Haynes, 2006, p. 140), were keen to both report independently and avoid encroaching on territory reserved to the MOH. The result is a style of reporting that reads most like the discursive introductory section of a MOH report; the dense statistics and detailed accompanying commentary of the later sections are largely absent from the FSI reports. Yet, MOsH and other experts in public health were not the sole point of reference. Scholars have drawn attention to the national influence of Octavia Hill’s work on the priorities, and reporting style, adopted by pioneer women public health officials (Harris, 2004, pp. 133–134; Hollis, 1987, p. 13). Significantly, the Bradford FSI reports were also both informed by, and part of, a long tradition of social enquiries in the UK between 1880 and 1914 (Welshman, 2006, p. 1; Marshall, 1970, pp. 24–45; Harris, 2004, pp. 57–58, p. 152). T. H. Marshall (1970, p. 26) records the ‘sensation’ created by a series of books documenting urban poverty, and the FSI reports were almost contemporaneous with Benjamin Seebohm Rowntree’s York investigations and the final volume in Charles Booth’s series on the poor in London. Marshall additionally notes how widely these publications were read and the impact they had on influential thinkers of the day. This favourable climate helps explain how and why the FSIs reported their work in the manner they did.

Like Rowntree, Booth, and other well-known investigators, the FSIs were interested in the causes and consequences of poverty. Their reports provided regular discussions about the state of local labour markets, wages, rents, prices and also special factors, such as old age, ill-health or severe weather, which presented particular difficulties. The value of such inquiries has been questioned by academics concerned by both the moralistic tone of such reports and the rather vague definitions of poverty that underpinned them (Townsend, 1975, p. 32, pp. 100–101; Welshman, 2006), but the insights offered by FSIs and others were welcomed and acknowledged by leading contemporary researchers. In the 1909 Minority Report of the Poor Law Commission, Beatrice Webb recorded that:

By the staff of health visitors and sanitary inspectors daily going their rounds, the Public Health Authority will become aware of cases in which the helpless deserving aged, notwithstanding their little pensions or the attentions of the charitable, are suffering from neglect or lack of care (Parker, 1965, 34 quotes Minority Report 1909: Part One, p. 353).

Intellectuals such as Sidney and Beatrice Webb were arguing that it was inefficient to think about the poor as a single entity. They advocated instead identifying specific groups with distinct needs and finding mechanisms to efficiently and effectively assist them without recourse to the Poor Law. The difficulties experienced by children, old people, the sick, and the unemployed were increasingly seen as issues that were distinct from pauperism. Such arguments fed into the work of the FSIs. Staff deliberately drew a personal and professional distinction between people they regarded as poor but decent, often struggling with impossible circumstances that were beyond their control but susceptible to relief if not remedy by intelligent state action, and other people whose personal conduct (though not necessarily economic situation) put them into a different category altogether. Here, ideas about mental defects were important. So too were theories about the residuum, which also connect with ideas articulated about the social problem group in the 1920s, and the problem family in the 1940s (Welshman, 2006).

## Ideas about mental defects and poverty

When the FSIs were talking about people and families who struck them as in some way different from the majority of slum dwellers they employed a number of prejudicial terms that reveal more about the class, gender and racial preoccupations of the authors, and the influence of the social purity movement, than the condition of the slums.

In a wretched little cottage…resided two married couples and two young children. At 11.30 am the occupants were still in bed and there was considerable difficulty in awakening anyone… [on gaining access FSI found] two dirty little children partially dressed were sitting by the fire; they both presented a very neglected appearance. They appeared to use the floor as a sanitary convenience, filth of all kinds being about. A slatternly woman crawled down a staircase which reminded one of an entrance to a hen roost, and there was very little furniture and only one bed. No windows being open, the air was most nauseating, and altogether one wondered whether this was a human habitation or a piggery (FSIR 31 March 1909, pp. 15–16).

These prejudicial terms were variously applied by FSIs to individuals, their homes, their observed and rumoured activities, and their alleged character failings; but a new coherence was offered by discussions about mental deficiency and its relationship to the residuum. Mathew Thomson notes that mental defectives:

[P]rovided a symbol which linked these overlapping anxieties about moral, demographic and racial decline: because of their mental defect they sank within society to join the residuum; lacking moral restraint they bred unchecked with similarly weak-minded individuals; and their offspring were brought up in such a socially and morally impoverished environment, and inherited such weak mental powers, that they perpetuated the vicious circle of decline, turning to crime or falling on the rates in order to survive (Thomson, 1998, p. 22).

The Bradford FSIs had plenty to say about mentally defective persons:

The feeble-minded wife has made no attempt to inculcate habits of decency in the children; the husband drinks and ill uses her (FSIR 26 March 1902, p. 5).

[In a case where the father was imprisoned for neglect, the mother detained in a workhouse and the children adopted by the Guardians] serious consideration should be given to the question whether the state might not be protected from the increase of the population through a stock inheriting viciousness from one parent and imbecility from the other (FSIR 31 March 1904, p. 7).

The mother was mentally deficient, the bedding and bedclothes were filthy, the bedroom was dirty and overcrowded and the children neglected. The matter was reported to the Guardians, and the mother removed to an asylum. An aunt came to superintend the home which is now clean and satisfactory (FSIR 29 September 1905, p. 8)

….the smell… added to the filth of the tenant, rendered the habitation almost unbearable to an ordinary person. The tenant was, however, mentally deficient, and unfortunately addicted to alcohol… (FSIR 30 September 1906, p. 8)

….woman working in a mill…home and children left to care of her ‘half-witted’ brother (FSIR 30 September 1907, pp. 11–12).

Staff confidently attached the label to various individuals whom they encountered, although there is no evidence that any of these people were known to authorities concerned with mental health and/or mental deficiency.

It was not just mental deficiency that interested the FSIs, but a whole range of apparently related problems. Anticipating the findings of the Royal Commission, which Thomson (1998, p. 32) notes came up with an entirely new classification system for all types of mental defect, the FSIs distinguished between conditions recognisable as ‘persons of unsound mind’ (the mentally ill), ‘persons mentally infirm’ (or senile), and various categories of mentally defective persons (including those additionally identified as inebriates or suffering from epilepsy or sensory disability). The FSIs were unhesitating when assigning people to these categories although mental health/deficiency topics are not prominent in discussions about training courses for Edwardian sanitary inspectors or health visitors (Dingwall et al., 1988, pp. 186–187; Haynes, 2006; Watts, 2007, pp. 147–148). It is quite possible that staff had no formal training in this field beyond possible attendance at one of the many public lectures in the West Riding of Yorkshire given by Mary Dendy and other activists (Jackson, 2000) or reading/listening to conference papers on the subject. This was not an uncommon practice as many educated lay people working across a variety of statutory and voluntary agencies asserted that their experiences, quite distinct from formal training, entitled them to claim expertise (Dale, 2006). However, in contrast to the paid and voluntary workers employed by the Central Association for the Care of Mental Defectives (Thomson, 1998, p. 153), set up to support the implementation of the 1913 Act, the FSIs were not tasked with identifying or supporting such individuals or their families. The attention given to the issue is inexplicable without contextualising this work within the wider study of poverty that the FSIs were engaged in as a personal and professional project.

Concerns about the deserving and undeserving poor are, of course, not confined to either the UK or this timeframe. Nevertheless, particular anxieties were undoubtedly raised around the turn of the last century by efforts to link, however tenuously, ideas about poverty, the residuum and new Darwinian theories that, for some, introduced a biological imperative to social action. Following Mathew Thomson’s detailed analysis, it is clear that that these ideas influenced the Royal Commissions on the Poor Laws and Mental Deficiency; although other issues were also considered and it is misleading to overstate the importance of any one factor or argument (Thomson, 1998, pp. 33–39). Contemporary critics were quick to point out that while mental defects were not confined to any one class of persons, state action was being deliberately targeted at the working classes, with emphasis on the poorest (Thomson, 1998, p. 46). The question of whether or not a ‘residuum’ existed has been hotly debated by historians (Thomson, 1998, p. 21), but there is certainly agreement that the term gained currency in the late nineteenth century and was frequently discussed in social inquires and parliamentary papers, and press coverage of both, in the period 1880–1914 (Welshman, 2006, pp. 1–20). The FSIs recognised and deployed this language (and related terminology), clearly finding it highly relevant to their experiences. They demonstrated a particular concern with preventing the residuum from dragging down the other slum dwellers whose poverty did not prevent them seeking the self-improvement that the staff aimed to encourage.

Schemes of visiting relied on the willingness and ability of clients to respond positively to the advice and instructions offered. On the whole the Bradford FSIs were pleased about the reception they received and optimistic about what could be achieved. In fact, it was the competence and resilience of the majority of the slum dwellers that impressed them, and indeed other contemporary social investigators. Patricia Hollis (1987, p. 23) notes that studies tended to reveal ‘that destitution was less a problem of the inadequate mother, work-shy father and demoralised family, as the under-paid father, the large family, and the exploitative workplace’ (with the Bradford FSIs also keen on a narrative of neglectful and exploitative landlords and the failure of wealthier citizens to support necessary statutory and voluntary sector programmes). Yet nationally, prominent women contributors to these debates persisted with a focus on character and behaviour that for some ruled out accepting ‘structural explanations of poverty and collective solutions to it’ (Welshman, 2006, p. 17). The Bradford FSIs, caught in the ideological contradictions already discussed, explained most of the poverty they witnessed in terms of low wages and inadequate resources, but also highlighted a distinct subset of cases where something very different was believed to be going on.

Most of the cases identified were framed by contemporary concerns about mental deficiency, although their presentation is in many ways identical to much later descriptions of problem families (Welshman, 1996; Starkey, 1998), where poor relationships between family members and between the family, its neighbours and officialdom were seen as at least as problematic as a lack of money or the functioning of any one individual. The FSI reports provide an official verdict on the homes visited and there is no voice for clients. However, oral histories conducted at a later date offer some insights into the painful isolation, as well as disorganised chaos experienced by families whose behaviour (especially around drinking, violence and bad language) made them uncongenial to fellow slum dwellers, as well as targets for state and voluntary sector interventions. Thea Thompson (1981, pp. 9–12, pp. 13–35) used the memories of Londoner ‘Thomas Morgan’ to illustrate these points, but notes that despite her struggles the matriarch of the Morgan family still maintained ‘some of the habits of a respectable woman’. Women public health officers, especially in the guise of health visitors, typically represented themselves as the ‘mother’s friend’ (Davies, 1988). This was an arrangement that allowed ‘social control’ to be ‘exercised invisibly through the desire to maintain a relationship with a representative of authority that confers approval and advertises respectability’ (Dingwall, Rafferty and Webster, 1988, p. 188).

## The problem of respectability encouraging silence on mental health topics

Respectability was a powerful concept that impacted on the expectations and behaviours of visitors and visited alike, and the relationship between them. Although most commentators have concentrated on the woman-to-woman contacts believed to be at the heart of health visiting practice, the FSIs’ primary concern with accommodation issues meant that their remarks were often addressed to male householders, as well as housewives. The problem was that in overcrowded homes and districts, offering a minimum of privacy, the quest for respectability often directly conflicted with health and hygiene messages. A reluctance to publicly dispose of kitchen slops and human excrement certainly encouraged problems with smells, and spread disease (Thompson, 1984). Fresh air was advocated by health promoters but responsible mothers refused to let their children wander to healthful local parks despite risking contamination (moral, as well as physical) by keeping them close to slum homes. Respectable people sent their children to school, but such institutions were an important locus in the spread of infections. Respectable people also shunned money-making activities, such as taking in lodgers, or engaging in informal trading or breeding animals or birds, that would have added to the household budget and allowed the family a better standard of nutrition or, better still, a move out of the worst slum areas. They also avoided charitable aid, preferring to suffer in silence. The FSIs were dismayed to record:

This winter has been peculiarly trying, owing to the great amount of destitution. On one of the coldest days, when the snow was falling heavily, one of the most respectable of our mothers was found to be absolutely without food or fire. During the daytime she spent her time with a kind neighbour, who gave her shelter, but at night, she returned to their miserable home and was obliged to undress the little ones without any fire in the grate; this was, unfortunately not an isolated incident (FSIR 31 March 1909, p. 11).

Tensions between promoting respectability and protecting physical health have been widely discussed by contemporary and historical commentators concerned with slums (Thompson, 1984), but the FSIs went further than this by considering mental well-being. As the FSIs became more knowledgeable about, and sympathetic towards, slum dwellers they looked again at behaviours they had originally simply condemned. As conscientious promoters of thrift and sobriety, the FSIs disapproved of the public house and drinking, as well as drunkenness (alcoholism always attracted their harshest criticism). But, they came to understand how the light, warmth and, perhaps especially, companionship offered by the pub appealed to those whose homes were cold, dark and drab (FSIR 30 June 1908, p. 6). Social networks have long been understood to be central to the economic survival of slum dwellers (Harris, 2004, p. 77). But, loneliness as a cause and consequence of what we would now term depression and even dementia was a problem at least implicit in FSI discussions of neglected older people (FSIR 30 September 1906, pp. 8–9), struggling young mothers, and vulnerable recent immigrants with no local contacts and language and other barriers to overcome before settling into an area. Recognising the importance of having kinship and community links, that had emotional, as well as economic value, helped explain people’s attachment to the slum. It also made understandable their fears about leaving, especially when economic insecurity made it almost certain that any escape would be temporary.

The FSI reports certainly developed themes that resonate with twenty-first-century discussions about mental health. However, such was the stigma of mental illness that FSIs do not appear to have discussed mental health/ill-health directly with the people they visited. A particular silence attached to women’s health issues due to their close association with reproduction and gynaecological problems (Gittins 1982, p. 42). This silence also arguably extended to women’s mental health. In a really interesting discussion about the under-representation of certain groups of women in Brookwood Asylum admissions, Anna Shepherd (2014, pp. 94–95) hypothesises about the lack of time poor women would have had to consider their psychological problems and the difficulties working-class women would have experienced when seeking a medical consultation to address them. She also suggests that women with conditions that were not too severe were kept at home by their families rather than being sent to an asylum because they were not dangerous and could still perform useful domestic tasks.

The poor mother, quick to divert scarce household resources to both the male breadwinner and her children, was however particularly vulnerable to developing the chronic physical health problems that pre-1914 asylum doctors recorded in so many of their patients. This point concerned the FSIs, whose anxiety about the welfare of the mother extended to the unborn child and the future of the race. Pregnancy was seen to imperil the physical health and emotional balance of the mother, with poverty a major complication and significant source of stress:

In most cases where the wages are inadequate, the mother is the first to deny herself, and in the case of child-bearing women, this has the most serious results. In the homes, it is often noticeable that in times of stress and difficulty, the children are fed first, then the father, and the mother has what remains, or goes without. Women are naturally more unselfish than men, their training makes them so, and it is ever the woman who suffers most from poverty (FSIR 1 October 1909–1930 September 1910, p. 11).

The poor state of the bodies and clothing of new asylum admissions tended to be related less to neglectful prior care and more to the serious impact that deprivation was understood by asylum doctors to have on mental health. Anna Shepherd (2014, p. 81) usefully records one superintendent’s thinking on the impact of a trade depression and connections between poverty and insanity. When considering individual cases, however, money worries and business anxieties were more likely to be ascribed to male patients. In the vast majority of pre-1914 asylum admissions the cause of insanity was listed as unknown, although historians have explored gendered patterns of diagnosis and treatment that provide some context to the work and thinking of FSIs.

Nineteenth-century asylum records list some of the known risks to mental health (Melling and Forsythe, 2006, p. 181, table 9.2). Some of these were closely connected to the community work of the FSIs, others less so. The FSI reports reveal they were alert to heredity, epilepsy, childbirth (though not menopause), ill-health/accidents, intemperance, grief/death, overwork and troubles/worry (including money). They were keen to talk about these issues in relation to the health of individuals and the functioning of families. The FSIs, however, had nothing particular to say about the impact religion, disappointments in love, over-study or fright might be having on the mental health of people they visited.

All these factors were, however, of some relevance to the FSIs own situation. As single women making their own way in the world it was essential for the FSIs to protect their own physical and mental health. This was difficult, though, as their work was physically demanding, and exposed them to environmental hazards and infectious disease. These dangers were well understood, and openly acknowledged. Threats to mental well-being were, perhaps, more insidious. The FSIs routinely had to deal with difficult people, and often had to confront distressing situations that could provoke primitive human fears (Bourke, 2005). While dealing with these professional problems the personal lives of the staff were restricted by a strictly enforced marriage bar that denied them the companionship of husband and children (Haynes, 2006, pp. 93–115). Stress in the caring professions is now an almost ubiquitous topic, and the distinctive social and economic problems experienced by female nurses and educators are associated with their visibility amongst admissions to pauper, and particularly private, asylums before 1914 (Melling, 2006).

The professional difficulties and personal frustrations of the FSIs probably complicated their relationship with clients and increased obstacles to a meaningful discussion about mental health. An additional problem was the way that personal experiences had to cover significant gaps that existed in both the mental health training the FSIs received and the limitations of other sources of information available to them. Thus, while the FSIs recorded really poignant deathbed scenes (involving deaths in childbirth and particularly TB cases), and made much of the grief of the families left behind, they were remarkably insensitive to other losses. The pain associated with miscarriage, still-birth or neonatal death was beyond the comprehension of spinster FSIs who simply recorded the total number of such events that midwives informed them about (Dale and Fisher, 2009). Similarly, when investigating infant deaths the FSIs focussed on their workload and personal distress. They failed to mention any anguish suffered by parents, although the deaths of children (even toddlers) attracted more thoughtful remarks. The FSIs showed real empathy with the agonies of parents who knew their child was sick (even dying) but were unable to secure timely medical assistance. Noting: ‘Adults, often in great distress of body and mind, may now wander vainly about the city in search of a recommend which can alone procure them the required medical aid’ (FSIR 30 September 1908, p. 6).

These problems of communication and understanding were exacerbated by the way services were organised. Before 1929, MOsH and their staff typically had very limited practical contact with workhouse or asylum care of the insane, although their professional publications made some references to the latest thinking in the field.^4^ Significantly, the FSIs had no official role in either the admission or discharge of patients and there is no mention of any Bradford scheme to follow up former patients. The FSIs themselves asserted that ‘the care of lunatics’ formed no part of their duties and bemoaned the fact that such cases were reported to them by members of the public who also expected the FSIs to concern themselves with ‘the morals of the people, matrimonial disagreements, [and] piano-playing’ (FSIR 30 September 1908, p. 8). In cases where the FSIs encountered people experiencing mental health crises (either accidentally or when summoned by concerned neighbours) they simply called for the urgent assistance of the Poor Law authorities. Thus one FSI reported:

I found the occupier to be a tall, haggard-looking woman, who appeared to be suffering from sickness. There was no fire in the grate and a most unpleasant odour emanated from the bedroom and the person of the tenant. She complained to me of people endeavouring to poison her, and of faces appearing at the window. I thought her to be mentally deranged, and, promising to return shortly, I made inquiries with reliable sources… The Guardians kindly made arrangements to have her medically examined and removed to the workhouse infirmary (FSIR 30 December 1096, pp. 5–6).

Some of these cases (especially involving alcohol and domestic violence) and incidents clearly distressed, even frightened, the FSIs. They routinely faced many hazards in the course of their work, but sought to avoid contact with dangerous individuals by recording where they lived and making special arrangements if they needed to be visited.

The unhelpful conflation of mental health issues with mental health emergencies, dangerous people, and risks to staff arguably detracted attention away from the less severe, but more common, mental health problems that the FSIs were likely to encounter. There were fleeting references to meeting ‘fearful’, ‘nervous’ and ‘anxious’ people and people whose behaviour was more than eccentric,

A curious feature of this case was the discovery of a man hiding in the cellar (FSIR 25 December 1905, p. 5).

On investigating further a woman was found hiding in the cellar, and upstairs the mistress of the house was found to be in bed, having hurriedly hidden under the bedclothes, the sole evidence of her presence being a pair of boots protruding from the bottom of the bed (FSIR 25 March 1906, p. 9).

but no systematic discussion developed linking slum life to mental health problems. Indeed, it was professionally and politically necessary for the Bradford FSIs to emphasise the resilience of the slum dwellers and their capacity for self- and collective improvement. Those slum dwellers who did exhibit mental health problems (and more particularly mental deficiency) were regarded as a different problem, even a class apart. Such thinking chimed with developments in the asylum world, where a ‘hereditary taint’ was used to explain an increasing number of cases between 1900 and 1914 (Cherry, 2003, pp. 125–126), but it ran counter to other patterns in asylum admissions. Steven Cherry makes the interesting point that bodily disease, old age and alcoholism remained important markers on male patients’ journey to the pre-1914 asylum. These issues applied to women as well, although in their case ‘domestic’ causes and ‘stress’ formed distinct categories in the admission registers (Cherry, 2003, p. 129).

All too aware of the routine drudgery and periodic crises of working-class women’s lives (Gittins 1982; Ross, 1993), the FSIs were well-placed to comment on these issues,

During the year [the lady mayoress] kindly gave two meat teas to our poorest mothers; the tea was most daintily served and gave an immense amount of pleasure to these women, whose lives are very grey, and often nothing but drudgery. After tea, they were provided with an entertainment which considerably added to their pleasure, and many of them still refer to the event as one of the brightest in their lives. It also helped to encourage them, as they realised their more fortunate sisters took an interest in the welfare of themselves and their little ones (FSIR 31 March 1909, p. 11).

Yet, apart from the significant occasion above chose not to. This was arguably because severe mental illness was so uncommon in their daily work, despite all the obvious threats to physical, mental and moral health from the slum environment. Asylum superintendents had independently considered this point. Late-Victorian asylums were full and demand for their services was growing, but given the hazards of urban and industrial life the real question for thoughtful commentators was: why were not more people becoming insane? As early as 1889 David Thompson, at the Norfolk asylum, hypothesised ‘intemperance, childbirth, ill-health, domestic trouble and anxiety can only produce insanity in minds predisposed to it by hereditary taint, for surely these causes are rife around us without causing insanity’ (Cherry, 2003, p. 130).

## Discussion

At the start of the twentieth century public health provision in the UK was moving away from its origins in nineteenth-century sanitary reform, towards new models of personal and preventative health services. These reforms required new staff and innovative ways of engaging with targeted client groups. It was the woman-to-woman conversation at the heart of so many of these initiatives that excited contemporary commentators such as Dr Bostock Hill (Dingwall et al., 1988, p. 187) and attracted the attention of later generations of social historians and feminist scholars. Their sophisticated critiques of the aims, methods and achievements of pioneer visiting schemes (conducted by health visitors and/or other female staff) have usefully highlighted class and gender relations as both organising points and significant sources of tension. Poverty and its management emerge as key themes from the historiography, but such analysis can crowd out other issues. The literature is, for example, almost totally silent on the relationship that contemporary actors drew between poverty and mental health.

The records created by the Bradford FSIs provide powerful descriptions of the acute and enduring poverty experienced by slum dwellers. They also explore the damage staff believed such poverty was inflicting on individuals, families, and communities. Initially, FSI discussions centred on immediate threats to physical health, but over time mental health and well-being emerged as distinct concerns. Such interests and activities were closely bound up with professional projects. This was a moment when women sanitary inspectors were fighting a defensive (and ultimately losing) battle over their reinvention as health visitors. Mental health and mental deficiency were fashionable topics and staff (and their professional leaders) hoped to bolster their professional credentials by demonstrating relevant, even unique, expertise. If women officers could escape a narrow concern with women and infants, they would be better placed to maintain parity with male sanitary inspectors and preserve their valued tradition of independent reporting. The Bradford FSI reports served to advance these agendas. They also anchored the work of the FSIs in the wider public health movement. Perhaps most significantly, they allowed the staff to communicate with groups engaged in ongoing political battles over the future of municipal services in the city. The distinctive contribution of the Fabian Society to reforms in Bradford and the national development of professions such as sanitary inspection (Haynes, 2006, p. 59) should not be underestimated.

The case study encourages a broader re-evaluation of histories of mental health care in so far as they discuss relationships to poverty and relationships between service-providers and serviceusers. Pre-1914 asylum studies foreground poverty as a cause and consequence of mental illness in ways that histories of mental health care throughout the rest of the twentieth century, far more concerned with tracking changing diagnostic labels and therapeutic innovations, do not always manage to replicate. In part, this is because of the proliferation (and fragmentation) of professional, and increasingly lay, voices that claimed relevant knowledge and expertise. The Bradford FSIs serve as an early example of how new professional groups inserted themselves into the field of mental health. Remarkably, the work of the Bradford FSIs was based solely in the community. Many historians highlight the hostility of clients to visits. Yet, there is ample evidence that, when confronted with mental health crises, slum dwellers actively sought the assistance of FSIs. In, fact in these scenarios, it was most often the officer rather than the client who was reluctant to proceed. Several questions are thus provoked: why did the slum dwellers seek this assistance, and what did they hope or expect would result from it? Perhaps, either imperfect knowledge of, or frustration with, existing models of service delivery and/or arrangements for accessing provision explain much. Such concerns, hitherto unexplored in the literature, merit further consideration as they illuminate debates about mental illness and mental health care being conducted in locales and between actors not usually considered. It is significant that poverty was such an overarching but also contentious theme.

### Data sharing

Data sharing not applicable to this article as no datasets were generated or analysed during the current study.

## Footnotes

1 For example, the FSI reports cited and sometimes summarised papers given by different actors at major conferences (FSIR 31 March 1908, pp. 7–8) and also mentioned various inquiries and reports and even press coverage of certain issues.

2 Local history sources are helping to build a more nuanced picture of the lives and careers of pioneer sanitary inspectors such as Mary Leslie (nee Martin), the first lady sanitary inspector appointed in Stockton-on-Tees in 1901. See www.stockton.gov.uk/people/mary-leslie-inspector-of-sanitary-in-stockton-working-classes/. Accessed 29 Jan 2018

3 The Cinderella Club was a charity aiding necessitous mothers and babies. It was a major provider of milk to nursing mothers and humanised milk to infants in Bradford (FSIR 31 March 1908, p. 8) before municipal schemes were introduced in 1911. By the 1920s the charity was primarily concerned with providing clothes and toys to poor children and also supported a holiday home (Bradford Council of Social Service 1923, p. 158).

4 A noteworthy exception perhaps being Exeter where embryonic World War One infant welfare schemes depended on the services of the doctor otherwise employed as medical superintendent at the city asylum.

## References

[R1] Adams C (2012). The history of health visiting. Nursing in Practice.

[R2] Bourke J (2005). Fear: a cultural history.

[R3] Bradford Council of Social Service (1923). The texture of welfare: a survey of social service in Bradford.

[R4] Bradford Local Studies Centre (1902-1911). City of Bradford Central Library, B614 FEM, Reports of the work of the female sanitary inspectors (FSIR).

[R5] Brimblecombe P (2000). Historical perspectives on health: the emergence of the sanitary inspector in Victorian Britain. J R Soc Promot Health.

[R6] Cherry S (2003). Mental Healthcare in Modern England: The Norfolk Lunatic Asylum/St Andrew’s Hospital c. 1810-1998.

[R7] Clark J (1973). A family visitor: a descriptive analysis of health visiting in Berkshire.

[R8] Council for the Education and Training of Health Visitors (1982). Health visiting principles in practice.

[R9] Dale P, Dale P, Melling J (2006). Tensions in the voluntary-statutory alliance: ‘lay professionals’ and the planning and delivery of mental deficiency services, 1917–1945. Mental Illness and Learning Disability Since 1850: Finding a Place for Mental Disorder in the United Kingdom.

[R10] Dale P (2008). The Bridgwater infant welfare centre, 1922-1939: From an authoritarian concern with ‘welfare mothers’ to a more inclusive community health project?. Fam Community Hist.

[R11] Dale P, Fisher K (2009). Implementing the 1902 Midwives Act: assessing problems, developing services and creating a new role for a variety of female practitioners. Women’s Hist Rev.

[R12] Dale P, Mills C (2007). Revealing and concealing personal and social problems: family coping strategies and a new engagement with officials and welfare agencies c. 1900–1912. Fam Community Hist.

[R13] Davies C (1988). The health visitor as mother’s friend: a woman’s place in public health, 1900-1914. Soc Hist Med.

[R14] Davin A (1978). Imperialism and motherhood. Hist Workshop J.

[R15] Dingwall R (1977). The Social Organisation of Health Visitor Training.

[R16] Dingwall R, Rafferty AM, Webster C (1988). An introduction to the social history of nursing.

[R17] Dwork D (1987). War is good for babies and other young children: a history of the infant and child welfare movement in England, 1898-1918.

[R18] Dyhouse C (1979). Working-class mothers and infant mortality in England, 1895-1914. J Soc Hist.

[R19] Francis H, Warren M, Francis H (1987). Public health: the decline and restoration of the tradition. Recalling the medical officer of health: writings by Sidney Chave.

[R20] Gittins D (1982). Fair Sex: Family Size and Structure, 1900-39.

[R21] Halifax Local Studies Centre (HLSC), 352 Hal, Halifax County Borough Council minutes.

[R22] Harris B (1995). The health of the schoolchild: a history of the school medical Service in England and Wales.

[R23] Harris B (2004). The origins of the British welfare state: social welfare in England and Wales, 1800-1945.

[R24] Haynes JR (2006). Sanitary Ladies and Friendly Visitors: Women Public Health Officers in London 1890-1930.

[R25] HLSC (1900-1974). 614 Hal, County Borough of Halifax, Report of the Medical Officer of Health.

[R26] Hollis P (1987). Ladies elect: women in English local government 1865-1914.

[R27] Howells JG, Cunningham PJ (1967). Psychiatric aspects of the family and Special aspects of psychiatry. The principles of health visiting.

[R28] Jackson M (2000). The borderland of imbecility: medicine, society and the fabrication of the feeble mind in late Victorian and Edwardian England.

[R29] Kelly A, Symonds A (2003). The social construction of community nursing.

[R30] Laybourn K (1994). The Guild of Help and the changing face of Edwardian philanthropy: the guild of help, voluntary work and the state, 1904–1919.

[R31] Levene A, Powell M, Stewart J, Taylor B (2011). Cradle to grave: municipal medicine in interwar England and Wales.

[R32] Lewis J (1984). Women in England 1870-1950: sexual divisions and social change.

[R33] Lewis J (1986). Labour and love: women’s experiences of home and family, 1850–1940.

[R34] Marland H (1993). A pioneer in infant welfare; The Huddersfield scheme 1903–1920. Soc Hist Med.

[R35] McIntosh T (1998). Profession, skill or domestic duty? Midwifery in Sheffield, 1881–1936. Soc Hist Med.

[R36] Mckeown T, Lowe CR (1974). An Introduction to Social Medicine.

[R37] Malone M (2000). A history of health visiting and parenting in the last 50 years. Int Hist Nurs J.

[R38] Marshall TH (1970). Social policy in the twentieth century.

[R39] Melling J, Forsythe B (2006). The Politics of Madness: The State, Insanity and Society in England, 1845–1914.

[R40] Newsholme A (1936). The last thirty years in public health: recollections and reflections on my official and post-official life.

[R41] Nottingham C, Dougall R (2007). A close and practical association with the medical profession: Scottish medical social workers and social medicine, 1940–1975. Med Hist.

[R42] Owen G (1977). Health visiting.

[R43] Parker J (1965). Local health and welfare services.

[R44] Ross E (1993). Love and toil: motherhood in outcast London, 1870–1918.

[R45] Shepherd A (2014). Institutionalizing the insane in nineteenth-century England.

[R46] Starkey P (1998). The medical officer of health, the social worker and the problem family 1943 to 1968: the case of the Family Service Units. Soc Hist Med.

[R47] Thompson B, Woods R, Woodward J (1984). Infant mortality in nineteenth-century Bradford. Urban Disease and Mortality in Nineteenth-Century England.

[R48] Thompson T (1981). Edwardian childhoods.

[R49] Thomson M (1998). The problem of mental deficiency: eugenics, democracy and social policy in Britain c. 1870–1959.

[R50] Thomson M (2006). Psychological subjects: identity, culture and health in twentieth-century Britain.

[R51] Townsend P (1975). Sociology and Social Policy.

[R52] Vincent D (1991). Poor citizens: the state and the poor in twentieth-century Britain.

[R53] Watts R (2007). Women in science: a social and cultural history.

[R54] Welshman J (1996). In search of the “problem family”: public health and social work in England and Wales, 1940-70. Soc Hist Med.

[R55] Welshman J (1997). Family visitors or social workers? Health visiting and public health in England and Wales 1890-1974. Int Hist Nurs J.

[R56] Welshman J (2000). Municipal medicine: public health in twentieth-century Britain.

[R57] Welshman J (2006). Underclass: a history of the excluded 1880–2000.

